# The regulatory effect of *Lactobacillus rhamnosus GG* on T lymphocyte and the development of intestinal villi in piglets of different periods

**DOI:** 10.1186/s13568-020-00980-1

**Published:** 2020-04-17

**Authors:** Seria Masole Shonyela, Bo Feng, Wentao Yang, Guilian Yang, Chunfeng Wang

**Affiliations:** 1 College of Animal Science and Technology, Jilin Provincial Engineering Research Center of Animal Probiotics, Jilin Agricultural University, 2888 Xincheng Street, Changchun, 130118 China; 2 Ministry of Agriculture, Livestock and Fisheries, P.O. Box 9152, Veterinary Complex, 131 Nelson Mandela Rd, Dar es Salaam, Tanzania

**Keywords:** Piglet, Immune function, *Lactobacillus rhamnosus*, P40 protein, T lymphocytes

## Abstract

The maturation and development of T cells were not completed until T cells were selected in thymus. It was not until the early 1960s that j.f.a.p. discovered the importance of thymus in T cell development. Twelve healthy piglets were randomly divided into two groups, the experimental group (LGG group) and the control group (saline group). The LGG group piglets were given 1 ml LGG (6 × 10^9^ CFU/ml) per day. The saline group was given 1 ml of normal saline per day. The piglets were slaughtered at 30 days and 45 days, respectively, and the MLN, jejunum and ileum PPs, LP of the piglets were taken. The expression of CD3^+^CD4^+^ T lymphocytes was detected by flow cytometry, and intestinal villi development was observed by intestinal paraffin section. The results showed that the flow cytometry results at 30 days and 45 days showed that the CD3^+^CD4^+^ T lymphocytes in the MLN group were significantly different from those in the saline group (P < 0.05, P < 0.01).The CD3^+^CD4^+^ T lymphocytes in the jejunum PP of piglets in LGG group were significantly different from those in saline group (P < 0.05). The CD3^+^CD4^+^ T lymphocytes in the ileum PP of the LGG group were significantly different from those in the saline group (P < 0.05, P < 0.01). CD3^+^CD4^+^ T lymphocytes and normal saline in the piglets of the LGG group There was a significant difference between the two groups (P < 0.001, P < 0.05). P < 0.001). HE staining results showed the length of the LGG group ileal villi in piglets at 30 days, 45 days was significantly different from that in normal saline group (P < 0.01, P < 0.01). LGG can also regulate the proliferation of T lymphocytes in the intestine of early weaned piglets at 30 days and 45 days increase the number of CD3^+^CD4^+^ T lymphocytes.

## Introduction

The maturation and development of T cells were not completed until T cells were selected in thymus. It was not until the early 1960s that j.f.a.p. discovered the importance of thymus in T cell development. Miller, an Australian biologist, tried to expand his view that thymus is the place where cells develop. It is an underrated organ, and thymocytes are small, thin, dim and ineffective non characteristic cells (Russler-Germain et al. 2017). However, Miller has proved that thymus is an important part of T lymphocyte maturation. T cell precursors still retain the ability to provide a variety of hematopoietic cell types, which are transmitted from the bone marrow to the thymus through the blood. They are called thymocytes because of their mature sites (López et al. 2012). When immature T cells mature into functional T cells, they pass certain developmental stages in specific thymic microenvironment. Thymus is a special environment in which immature T cells produce a unique antigen receptor (T cell receptor or TCR), and then select according to their reactivity to the self MHC peptide complex expressed on the surface of thymic stromal cells. Thymocyte T cell receptor binding to its own MHC peptide complex has a high affinity to be induced to die (negative selection), and those thymocytes binding to their own MHC peptide have a positive selection, leading them to survive, mature and migrate to thymic medulla. Most thymocytes can’t pass through thymus successfully. In fact, it is estimated that 95% of thymocytes die during transportation. Most cell death is due to their low affinity for the self antigen MHC combination encountered on the surface of thymic epithelial cells and their inability to make positive selection (Lutter et al. 2018).

T cell development occurs in several different thymic microenvironments. T cell precursors enter the thymus in the blood vessels at the junction of the cortex and medulla between the thymus cortex (outside the organ) and the thymus medulla (inside the organ). At this stage, neither CD4 nor CD8 were expressed in thymocytes. As a result, they are called double negative (DN) cells. DN cells first enter the area under the thymic sac, known as the subcapsular cortex, where they proliferate and begin to produce their own T-cell receptors. Thymocytes that successfully expressed TCR began to express CD4 and CD8, becoming double positive (DP) cells, and filled the cortex, which was the site where most (85% or more) immature T cells were found. Cortex has a unique group of stromal cells, cortical thymic epithelial cells (ctecs), whose long process is detected by thymocytes, and the ability of T cell receptor to bind to MHC peptide complex is tested (Sujino et al. 2016). After selection, the surviving thymocytes moved to thymic medulla, among which the positive thymocytes met the specialized stromal cells, medullary thymic epithelial cells (MTEC). MTEC not only supports the final step of thymocyte maturation, but also has the unique ability to express proteins. Otherwise, proteins are only found in other organs (Ou et al. 2018). This allows them to negatively select a group of potentially very damaging self reactive T cells that cannot be removed from the cortex. Mature thymocytes express only CD4 or CD8 and are known as single positive (SP) cells that leave the thymus through blood vessels at the cortical medullary junction when they enter (Maeda et al. 2018). Mature at the periphery, these new T cells explore antigens present in secondary lymphoid tissues, including the spleen and lymph nodes.

T cells combine with peptides in MHC protein grooves to form complex antigens. When the T cell receptor contacts with MHC peptide antigen on the surface of antigen presenting cell, the two cell membranes are closely juxtaposed with each other (Schwarz et al. 2010). This adds an extra layer of complexity to the process of T cell activation. Despite this extra complexity, the process of T cell activation is still carried out precisely according to the above pathway, and has many similarities with B cell receptor signal transduction. Here, we briefly describe the structure of T cell receptor, and then turn to the characterization of signal transduction pathway through the receptor.

T cells have two types of receptors, both of which are heterodimers (dimers consisting of two different polypeptides). Most recycled T cells have α–β heterodimers, which bind to ligands composed of antigenic peptides present in molecular trenches on the surface of type I or type II MHC molecules (Bercovici et al. 1999). The second T cell subsets instead express heterodimer T cell receptors composed of different protein chains, called γ and δ T cells carry the γ δ receptor with a specific localization pattern (in mucosal tissue) and some γ δ T cells recognize antigens from T cells binding to different types of antigens. Although some T cells recognize peptide antigens presented by conventional MHC, other α–β T cells bind to lipid or glycolipid parts presented by nonclassical MHC molecules (Nielsen et al. 2017). Only two such molecules that recognize MHC peptide antigens are CD4^+^ and CD8^+^. Mature T cells can be divided into two groups according to their expression of CD4 or CD8 on plasma membrane. CD4^+^ T cells recognize peptides binding to class II MHC molecules, mainly as helper or regulatory T cells, while CD8^+^ T cells recognize antigens expressed on the surface of class I MHC molecules, mainly as cytotoxic T cells (Zhong and Reinherz 2005). The extracellular domains of CD4 and CD8 bind to the conserved regions of MHCII and mhci molecules, respectively. The binding of single MHC molecules by TCR and CD4 or CD8 co receptors enhances the affinity of T cells to their targets (Park et al. 2010). This kind of conjugation also makes the cytoplasmic domain of TCR/CD3 close to the corresponding common receptor, and it helps to activate T cells. Signaling via antigen receptors, even when combined with antigen receptors via CD4 or CD8, is not sufficient to activate T cells that were not previously in contact with antigens (naive T cells It includes SLP-76 and lat, as well as enzymes important in T cell activation, such as plc1 (Chen et al. 2007).

## Materials and Methods

### Materials

Main instrument as shown in Table 1.

**Table 1:** Main instrument

Equipment name	Production unit
SW-CJ-2FD Single-sided double clean bench	Shanghai Boxun
ME204 Electronic analytical balance	METTLER TOLEDO, Switzerland
CJJ78-1 Type magnetic heating stirrer	Shanghai Meixiang Instrument Co., Ltd.
HRLM-80 Full-automatic autoclave	China Haier Co., Ltd.
BCD-649WDCEType refrigerator	China Haier Co., Ltd.
G70D20CN1P-D2(S0) 20L Microwave oven	China Galanz
HERAcell 240i37 °C CO2 constant temperature incubator	United States Thermo Fisher
Innova 40RType constant temperature shaker	United States NBS
5804R Desktop large capacity refrigerated centrifuge	Germany Eppendorf
Flow cytometry(LSR-FORTESA)	American BD Company
Real-time PCR instrument	Applied Biosystems

### Strain

*Lactobacillus rhamnosus* GG (ATCC 53103) was preserved by the Institute of animal science and technology of Jilin Agricultural University (Jilin Province animal microecological preparation engineering research center), and purchased in the United States model culture stock.

### Experimental animals

Twelve 25 day old healthy piglets were purchased from the breeding farm of Jilin University. The piglets were randomly divided into four groups, three in each group, and fed with sterile drinking water and antibiotic free feed.

### Main reagents

Dithiothreitol (DTT), sodium chloride, 300 mesh screen, 600 mesh screen and anhydrous ethanol were purchased from Beijing chemical preparation company. Tween 80, neutral protease, mouse collagenase IV type I DNase I, and red blood cell lysate were purchased from Biyuntian Company. Sodium azide, Percoll cell separation solution, PHA, PMA and ionomycin were purchased from sigma company, RPMI-1640, PBS buffer without Ca^2+^, Mg^2+^, fetal bovine serum and 0.5% BSA were purchased from GIBCO company. Antibody pe-cy7-cd3 (581477), fitc-cd3 (559582), pe-cd4 (559586), Alexa flow-cd8 α (561475) were purchased from BD company. Fitc-cd8 β (1114) tcr-1 (12-14-90), apc-cy7 fluorescent second antibody (ls-c341848) were purchased from Bio Company.

Main instrument as shown in Table 1.

## Method

### Grouping of experimental animals

Twelve healthy piglets were randomly divided into two groups, six in each group. They were control group (normal saline group) and experimental group (LGG). The normal saline group was administrated with 1 ml of normal saline for 15 days. LGG group was administrated with LGG (10^9^ CFU/ml), 1 ml each time. The MLN, jejunal PPS, ileal PPS, ileal lamina propria (LP) and jejunal lamina propria (LP) of three piglets were obtained at 35 days and 40 days, respectively. The single cell suspension was prepared. The number of T lymphocytes was detected by flow cytometry. The jejunum was taken out, and the ileum was about 3 cm each, as for 10% formaldehyde solution.

### MLN, PPS single cell suspension preparation method

In the ultra clean platform, MLN and PPS were stripped with ophthalmic scissors and ophthalmic forceps (Autoclaved), and the excess fat was removed. Put the folded 200 mesh sterile filter screen into a sterile plate, and add 1 ml rpmi-1640 culture medium. Put the tissue into the filter screen, gently grind the end of sterile 1 ml syringe until it is fully ground, then suck the liquid into 1.5 ml EP tube, put it into the precooled centrifuge, at 4 °C, 2000 rpm, and centrifuge for 5 min. Then discard the supernatant to obtain lymphocytes. After washing twice with FACS buffer, the cells were resuspended with 1 ml pbs and counted by cell counting plate after dilution.

### Preparation of LP and IEL single lymphocyte suspension

Take the jejunum and ileum for 10 cm, remove the excess fat, place them in PBS^−/−^ precooled at 4 °C, cut the intestine longitudinally, and clean the intestine with PBS^−/−^ precooled until it is cleaned. Then, the intestine was cut into about 1 cm with ophthalmic scissors. Put the cut intestine tube into 5 ml of IEL separation solution, vibrate at 37 °C for 15 min at constant temperature (200R/min), and put the intestine tube on 200 mesh nylon filter screen to discard the waste liquid. Repeat. The filtrate was single cell suspension of IEL, which was collected, counted and stained. Put the remaining intestinal tube into 5 ml of the inherent layer of lymphocyte separation solution, vibrate at 37 °C for 45 min with constant temperature (200R/min); filter the intestinal tube into a 300 mesh sterile filter screen, discard the solid residue, put the filtrate into a 15 ml sterile centrifuge tube, centrifugate at 4 °C, 400×*g*, for 10 min, discard the supernatant and collect LP cells. Take a new 15 ml centrifuge tube, place 4 ml 80% isotonic Percoll solution at the bottom of the 15 ml centrifuge tube, and then re suspend LP cells with 7 ml 40% isotonic Percoll solution. The LP cells were drawn with a 1 ml syringe, spread on 80% isotonic Percoll solution slowly and evenly, and centrifuged at 2300 rpm for 20 min at room temperature. Suck out the cell layer with two layers of liquid surface, transfer it into a new 15 ml centrifuge tube, add PBS^−/−^ to wash twice, and count the cell count plate after dilution.

### Antibody staining

Take 100 μl (1 × 10^6^ cells) of the above cells, and put them into 1.5 ml centrifuge tube. At the same time, a blank control tube and a single positive tube were set up; 10 μl of diluted antibodies were added to the corresponding cells, fully mixed, and incubated at 4 °C for 30 min in dark; 1 ml PBS was added to wash twice, and the cells were resuspended with 300 μl facs solution; the cells were put into the flow tube through 300 mesh nylon membrane and detected on the computer.

### Bioinformatics and statistical analysis

The data were analyzed by Graphpad prism 5.0 software. Compared with the data of the two groups, the student’s t test was used; compared with the data of the two groups, the multiple comparison method in one-way ANOVA was used for analysis, and the difference was statistically significant (P < 0.05).

## Results

### Effect of LGG on the number of CD3^+^ CD4^+^ T lymphocytes in MLN of piglets (30 days)

**Fig. 1:**
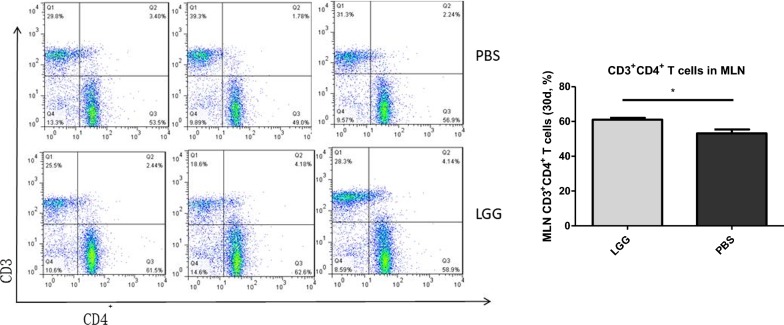
The number of CD3^+^CD4^+^ T lymphocytes in MLN administered with LGG at 30 days

The number of CD3^+^ CD4^+^ T lymphocytes in MLN of piglets was detected by flow cytometry. It can be seen from Fig. 1 that the number of CD3^+^ CD4^+^ T lymphocytes in MLN of piglets fed with LGG ATCC53103 (30 days) was significantly higher than that in normal saline group (P < 0.05).

### The effect of LGG on the number of CD3^+^ CD4^+^ T lymphocytes in MLN of piglets (45 days)

**Fig. 2:**
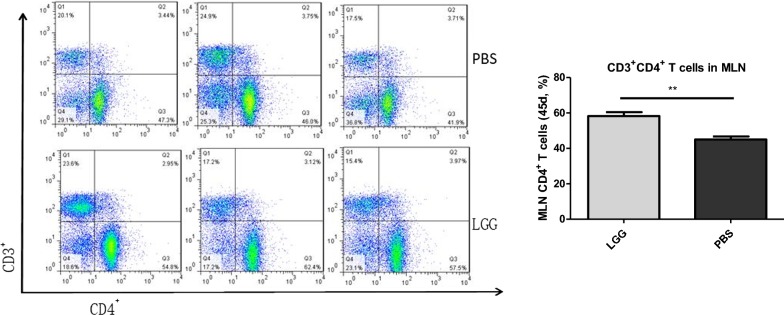
The number of CD3^+^CD4^+^ T lymphocytes in piglets MLN administered with LGG at 45 days

The number of CD3^+^ CD4^+^ T lymphocytes in MLN of piglets was detected by flow cytometry. It can be seen from Fig. 2 that the number of CD3^+^ CD4^+^ T lymphocytes in MLN of piglets fed with LGG ATCC53103 was significantly higher than that in normal saline group (P < 0.01).

### The effect of LGG on the number of CD3^+^ CD4^+^ T lymphocytes in PPS of piglets (30 days)

**Fig. 3:**
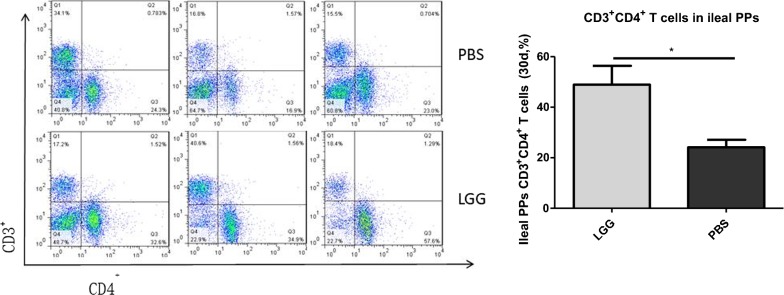
The number of CD3^+^CD4^+^ T lymphocytes in ileal PPs of LGG piglets at 30 days

We stained the single cell suspension of PPS in the ileum of piglets with antibody, and detected the number of CD3^+^ CD4^+^ T lymphocytes in PPS of piglets by flow cytometry. From Fig. 3 we can see that the number of CD3^+^ CD4^+^ T lymphocytes in PPS of piglets fed with LGG ATCC53103 (30 days) was significantly higher than that in normal saline group (P < 0.05).

### The effect of LGG on the number of CD3^+^ CD4^+^ T lymphocytes in PPS of piglets (45 days)

**Fig. 4:**
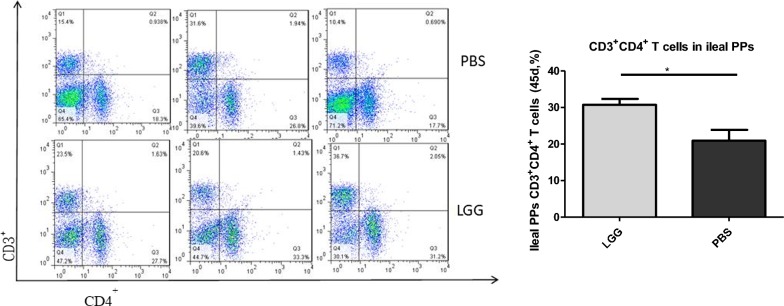
The number of CD3^+^CD4^+^ T lymphocytes in ileal PPs of LGG piglets at 45 days

The number of CD3^+^ CD4^+^ T lymphocytes in PPS of ileum of piglets was detected by flow cytometry. From Fig. 4, we can see that the number of CD3^+^ CD4^+^ T lymphocytes in PPS of ileum of piglets fed with lggatc53103 (45 days) was significantly higher than that in normal saline group (P < 0.05).

### The effect of LGG on the number of CD3^+^ CD4^+^ T lymphocytes in jejunal PPS of piglets (30 days)

**Fig. 5:**
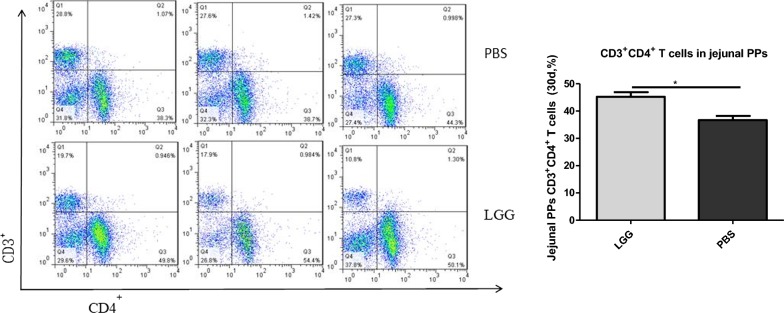
The number of CD3^+^CD4^+^ T lymphocytes in jejunum PPs of piglets fed with LGG at 30 days

The number of CD3^+^ CD4^+^ T lymphocytes in jejunal PPS of piglets was detected by flow cytometry. From Fig. 5, we can see that the number of CD3^+^ CD4^+^ T lymphocytes in jejunal PPS of piglets fed with LGG ATCC53103 (30 days) was significantly higher than that in normal saline group (P < 0.05).

### Effect of LGG on the number of CD3^+^ CD4^+^ T lymphocytes in jejunal PPS of piglets (45 days)

**Fig. 6:**
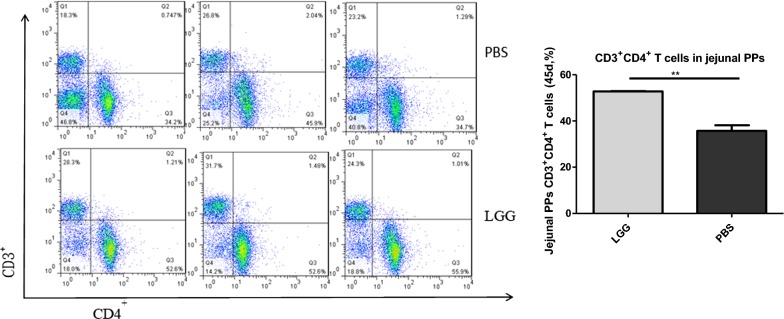
The number of CD3^+^CD4^+^ T lymphocytes in jejunum PPs of piglets fed with LGG at 45 days

The number of CD3^+^ CD4^+^ T lymphocytes in jejunal PPS of piglets was detected by flow cytometry. From Fig. 6, we can see that the number of CD3^+^ CD4^+^ T lymphocytes in jejunal PPS of piglets fed with LGG ATCC53103 (45 days) was significantly higher than that in physiological salt water group (P < 0.01).

### The effect of LGG on the number of CD3^+^ CD4^+^ T lymphocytes in piglet LP (30 days)

**Fig. 7:**
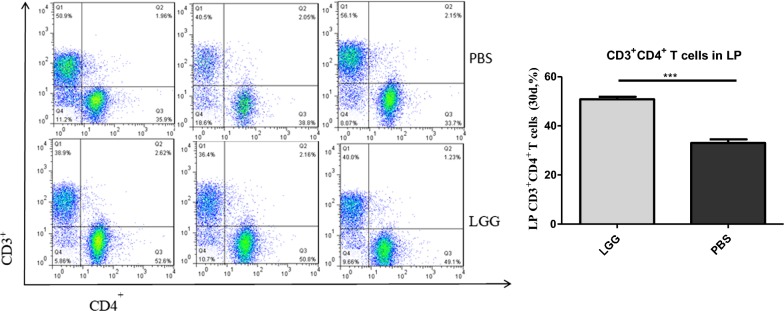
The Number of CD3^+^CD4^+^ T lymphocytes in PPs of piglets fed LGG at 30 days

The number of CD3^+^ CD4^+^ T lymphocytes in jejunal LP of piglets was detected by flow cytometry. From Fig. 7, we can see that the number of CD3^+^ CD4^+^ T lymphocytes in LP of piglets fed with LGG ATCC53103 (30 days) was significantly higher than that in normal saline group (P < 0.001).

### The effect of LGG on the number of CD3^+^ CD4^+^ T lymphocytes in piglet LP (45 days)

**Fig. 8:**
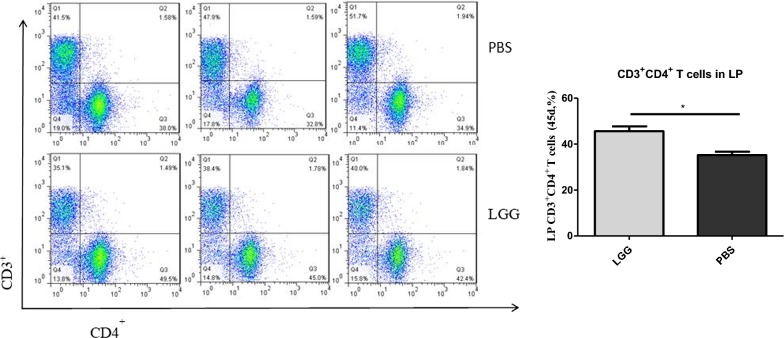
The number of CD3^+^CD4^+^ T lymphocytes in LP of piglets fed with LGG at 45 days

The number of CD3^+^ CD4^+^ T lymphocytes in jejunal LP of piglets was detected by flow cytometry. It can be seen from Fig. 8 that the number of CD3^+^ CD4^+^ T lymphocytes in LP of piglets fed with LGG ATCC53103 (45 days) was significantly higher than that in normal saline group (P < 0.05).

### Effect of LGG on intestinal villi in ileum of piglets (30 days)

**Fig. 9:**
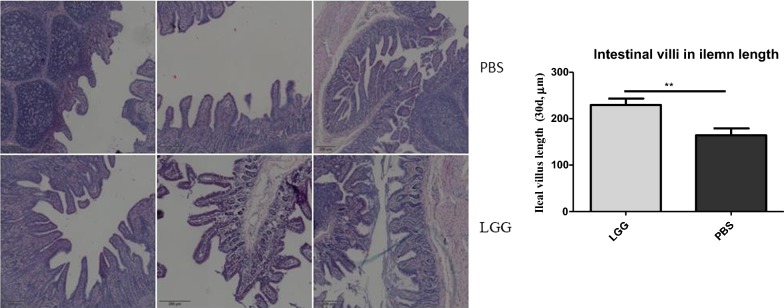
The HE-stained ileal sections of piglets at 30 days

He staining was carried out by paraffin section of the ileum of piglets. Results as shown in Fig. 9, the ileal villus length of piglets fed with LGGATCC53103 (30 days) was significantly higher than that of normal saline group (P < 0.01). The results showed that the intestinal villus integrity and development of piglets in LGG experimental group were better than that of normal saline group.

### Effect of LGG on intestinal villi of piglets (45 days)

**Fig. 10:**
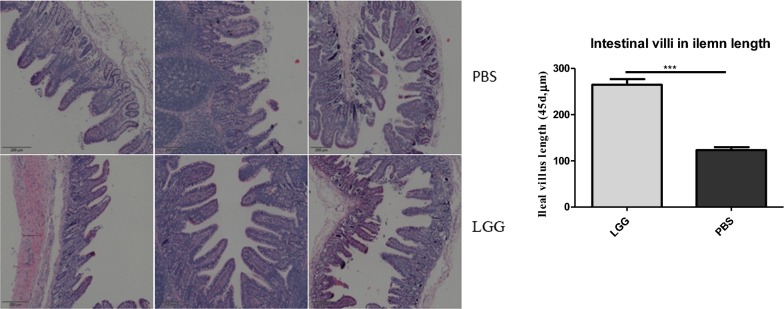
The HE-stained ileal sections of piglets at 45 days

He staining was carried out by paraffin section of the ileum of piglets. Results as shown in Fig. 10, the ileal villus length of piglets fed with LGG ATCC53103 (45 days) was significantly higher than that of normal saline group (P < 0.01).

Figure 10 the HE-stained ileal sections of piglets at 45 days.

### Effect of LGG on jejunal villi in piglets (30 days)

He staining was carried out on the paraffin sections of the ileum of piglets. Results as shown in Additional file 1: Figure S1, the length of jejunal villi of piglets fed with LGG ATCC53103 (30 days) was significantly higher than that of normal saline group (P < 0.01). The results showed that the integrity and development of intestinal villi in LGG experimental group were better than that in normal saline group.

### Effect of LGG on jejunal villi in piglets (45 days)

He staining was carried out by paraffin section of the ileum of piglets. Results as shown in Additional file 1: Figure S2, the length of jejunal villi of piglets fed with LGG ATCC53103 (45 days) was significantly higher than that of normal saline group (P < 0.01).

### Number of CD3^+^ CD4^+^ T lymphocytes in different tissues of LGG group at different time points

We compared the CD3^+^ CD4^+^ T lymphocytes in different tissues of LGG group at different time points, and the results are not shown. In MLN, LP, jejunum PPS and ileum PPS, there was no significant difference in CD3^+^ CD4^+^ T lymphocytes at any time point (P > 0.05). Therefore, we can prove that LGG has a long-term effect.

## Discussion

After consulting a large number of data analysis, because the MLN of piglets is not fully developed and does not start the effect of lymph homing (Tsuchida and Friedman 2017), so we set two different time points in this part to give the piglets LGG for 30 days and 45 days. On the one hand, we can determine whether the MLN of piglets fed with LGG for 15 days is not fully developed. On the other hand, we can verify that long-term LGG is not fully developed whether it can produce tolerance, whether it can still play a role in improving immunity. From the test results, we can see that the number of T lymphocytes in MLN of LGG group is significantly higher than that of normal saline group no matter on the 30th or 45th day. Therefore, we can confirm that our conjecture in the first chapter is correct. Similarly, we also detected the number of T-lymphocytes in other lymph nodes in the intestinal tract. The results of LGG group were significantly higher than those of normal saline group, which also proved that LGG can be taken for a long time and has been playing a role in immune regulation. Studies have shown that long-term use of LGG can prevent the occurrence of diarrhea, as well as the treatment of diarrhea has played a good effect. Some researchers have found that LGG can be transplanted into human intestine for a long time, from which it can regulate intestinal flora.

The incomplete morphological structure of intestinal mucosa is one of the causes of diarrhea in piglets. The development and integrity of intestinal villi can directly reflect the degree of stress injury suffered by the gut. A large number of experiments have proved that probiotics can effectively protect the normal development of intestinal villi (Esteban et al. 2013). Therefore, we also detect the integrity of intestinal villi by HE staining of intestinal paraffin section. From our results, it can be seen that the integrity of jejunum and ileum villi in LGG group is better than that in normal saline group at 30 or 45 days, and the length of villi is significantly longer than that in normal saline group, so we can conclude that LGG can protect the development and integrity of intestinal villi, It can protect intestinal villi.

At last, we compared three different time points of LGG group. There was no significant difference in the number of T lymphocytes in MLN, PP and LP. Previous experiments have proved that LGG can not produce tolerance to the body or cause flora disorder after long-term use, but can protect human intestinal tract for a long time and improve human immunity. We also got the same result. LGG can not tolerate piglets, but can protect the development of intestinal villi and regulate the immune function of piglets for a long time.

A large number of experiments have proved that probiotics can mediate human cellular immunity. Cellular immunity refers to the release of cytokines by T lymphocytes to mediate inflammation, so as to play an immune response. It was found that LGG can induce human T cells and dendritic cells to secrete cytokines (Ananta et al. 2005). It has been found that LGG can improve the proliferation of human T cells and increase the number of T cells (Khailova et al. 2013). Studies have shown that LGG can increase the number of T lymphocytes, thereby reducing the recurrence of human colitis (Mantegazza et al. 2017). In this experiment, we used LGG to administrate piglets in advance, and detected the number of T-lymphocytes in each lymph node and intestinal tract, as well as the differentiation of T-lymphocytes mediated by LGG. According to the analysis of the experimental results, there is no difference between the number of T-lymphocytes in MLN mediated by LGG and PBS group. We analyzed the reasons. As a secondary lymphoid organ, MLN circulates continuously in the whole blood and tissue fluid of T-lymphocytes. Because the ligand integrin released by the homing receptor MAdCAM-1 can make T-lymphocytes produce the homing effect, and we also looked up the results A large number of literatures showed that the development of MLN in piglets was slow. At the age of 35 days, MLN could not release a large number of ligands. In the following experiments, there was no significant difference in the number of T lymphocytes between the experimental group and the control group. So, in the next experiment, we will continue to verify this problem. We also analyzed the changes of the number of T-lymphocytes in other lymph nodes. From the results, we can know that LGG can induce the increase of the number of T-lymphocytes no matter in PP node or intestinal lamina propria, which also verified the results of previous studies. LGG has the function of inducing the proliferation of T-lymphocytes and increasing the number of T-lymphocytes, no matter in human or piglet It also provides a reference for the development of new Lactobacillus vaccine. T lymphocytes in the intestine can release a large number of cytokines to mediate intestinal inflammation. Because of this advantage of Lactobacillus, the new Lactobacillus vaccine will be paid more and more attention.

LGG can protect the development and integrity of intestinal villi, maintain the integrity of intestinal villi, and promote the growth of villi length. LGG can also regulate the proliferation of T-lymphocytes in the intestine of early weaning piglets at 30 days and 45 days, and increase the number of CD3^+^ CD4^+^ T-lymphocytes.

## Supplementary information

**Additional file 1: Figure S1.** The Piglets PP stained HE stained with LGG at30d. **Figure S2**. The Piglets PP stained HE stained with LGG at 45d.

## Data Availability

Not applicable.

## Abbreviations

BSA: Bovine serum albumin
DMSO: Dimethyl sulfoxide
EDTA: Ethylene diamine tetraacetic acid
PMA: Phorbol myristate acetate
FCM: Flow cytometry
LGG: *Lactobacillus rhamnosus* GG
MLN: Mesenteric lymph nodes
PPs: Pyle’s knot
LP: Lamina propria
Treg: Regulatory cell
IEC: Small intestinal epithelial cell
Th: T helper cells
TCR: T cell receptor
IL: Interleukin
IFN: Cervical lymph nodes
TGF: Transforming growth factor
