# Supplementary material for: The regulatory effect of Lactobacillus rhamnosus GG on T lymphocyte and the development of intestinal villi in piglets of different periods
Source: AMB Express. 2020 Apr 17;10:76. doi: 10.1186/s13568-020-00980-1 (PMC7165236; doi:10.1186/s13568-020-00980-1)
Supplement: Supplementary file 1 — Additional file 1: Figure S1. The Piglets PP stained HE stained with LGG at30d. Figure S2. The Piglets PP stained HE stained with LGG at 45d. [file 13568_2020_980_MOESM1_ESM.doc]

Additional Figure S1


Jejunal villi length

300	***


200


100


0


400

300
Jejunal villi length

 	*** 	


200

100

0
